# Unmasking Malignant Catatonia: A Diagnostic Challenge in a Febrile Patient With Altered Mental Status

**DOI:** 10.7759/cureus.108213

**Published:** 2026-05-04

**Authors:** Ying Huey Lim, Irene Yuen Lin Yii

**Affiliations:** 1 Internal Medicine, Singapore General Hospital, Singapore, SGP

**Keywords:** altered mental status evaluation, benzodiazepine use in catatonia, fever, hyponatremia, malignant catatonia, neuroleptic malignant syndrome (nms)

## Abstract

Malignant catatonia is a rare but life-threatening neuropsychiatric emergency characterized by catatonic features, autonomic instability, and fever. Prompt recognition is essential to avoid serious complications. We report a case of a 49-year-old Filipino male with schizoaffective disorder who presented with altered mental status and persistent fever. Initial investigations revealed severe hyponatremia, which was corrected, yet the patient remained encephalopathic. Despite an extensive workup, no infectious or structural cause was identified. He exhibited classic catatonic signs, including mutism, stupor, and echophenomena. A Bush-Francis Catatonia Rating Scale helps to support the diagnosis of malignant catatonia. Treatment with lorazepam led to rapid clinical improvement and resolution of fever. This case underscores the diagnostic complexity of malignant catatonia, particularly when presenting with fever and altered consciousness. Differentiation from neuroleptic malignant syndrome and encephalitis is critical due to differing management strategies.

## Introduction

Altered mental status is a frequent presentation in internal medicine, with a wide range of possible causes including primary central nervous system insults, systemic infections, metabolic disturbances, toxin exposure, medication effects, chronic systemic diseases, and psychiatric conditions [1]. Psychiatric conditions are typically diagnoses of exclusion and may contribute to delays in diagnosis.

Catatonia is a psychomotor syndrome that may arise in diverse psychiatric and medical contexts, with manifestations ranging from akinetic stupor to motor excitement [2]. Its most severe form, malignant catatonia, is characterized by fever, autonomic instability, and altered consciousness, and carries a mortality risk of approximately 10% if not treated promptly [3]. Malignant catatonia remains underdiagnosed in hospital practice [4].

Diagnostic challenges often occur because of its clinical overlap with other conditions, such as neuroleptic malignant syndrome (NMS), encephalitis, and metabolic encephalopathy, which can lead to delays in recognition [5]. Early diagnosis and intervention are essential, as malignant catatonia typically responds to benzodiazepines, with electroconvulsive therapy (ECT) reserved for refractory cases [3].

This case underscores the importance of recognizing and promptly treating malignant catatonia, as timely management can result in favorable outcomes. Malignant catatonia should be considered in the differential diagnosis when patients present with fever of unknown origin, dysautonomia [6], altered mental status, and mutism [7].

## Case presentation

A 49-year-old Filipino gentleman with a history of hypertension and schizoaffective disorder presented with altered mental status after collapsing at work. He had been on a stable dose of olanzapine and venlafaxine for the past year, with no recent dosage changes and good compliance.

On initial assessment, the patient was easily arousable to verbal stimuli but demonstrated poor verbal output. He appeared to track with his eyes. Speech assessment was limited due to reduced verbalization. No focal neurological deficit or significant unilateral weakness was observed. No seizure activity was observed. Laboratory investigations revealed severe hyponatremia with a serum sodium of 113 mmol/L (136-146 mmol/L). His presentation was attributed to symptomatic hyponatremia. Further history revealed that he consumed four to five liters of water daily, consistent with psychogenic polydipsia as the underlying etiology.

Vital signs were stable: temperature afebrile, blood pressure 110/75 mmHg, heart rate 96 beats per minute, and oxygen saturation 100% on room air. He was treated in the emergency department with 50 mL of intravenous (IV) 3% sodium chloride for symptomatic hyponatremia.

Initial laboratory investigations are summarized in Table 1.

**Table 1:** Initial laboratory investigations.

Investigations	Value	Normal range
Hemoglobin	13.4	12-18 g/dL
White cell count	12.33	4-10 x 10^9^/L
Lymphocytes	16.4	14-41%
Neutrophil	75.5	40-75%
Platelet count	417	140-440 x 10^9^/L
Urea	2.5	2.7-6.9 mmol/L
Sodium	113	136-146 mmol/L
Potassium	2.7	3.6-5.0 mmol/L
Chloride	76	100-107 mmol/L
Bicarbonate	18.5	19-29 mmol/L
Creatinine	62	54-101 µmol/L
Calcium	2.24	2.09-2.46 mmol/L
Phosphate	1.05	0.94-1.50 mmol/L
Total protein	69	68-85 g/L
Albumin	42	40-51 g/L
Bilirubin	24	7-32 µmol/L
Alkaline phosphatase	92	39-99 U/L
Alanine transaminase	68	6-66 U/L
Aspartate transaminase	54	12-42 U/L
Procalcitonin	0.1	<0.5 µg/L
C-reactive protein	2.6	0.2-9.1 mg/L

He was admitted to the high dependency unit for close monitoring. In view of overcorrection of sodium, he received two doses of 1 mcg desmopressin.

On day 2 of admission, he developed a fever without an obvious source. IV piperacillin/tazobactam 4.5 g every six hours was initiated according to hospital guidelines to provide empiric coverage for possible nosocomial infection. By day 3, his serum sodium had normalized to 135 mmol/L. Despite correction of sodium and antimicrobial therapy, he remained encephalopathic with intermittent fever. Subsequently, antimicrobial therapy was escalated to IV meropenem 1 g every eight hours to broaden coverage for suspected meningoencephalitis. Lumbar puncture was performed to exclude encephalitis, but the results were unremarkable. MRI brain and EEG were normal.

**Figure 1:**
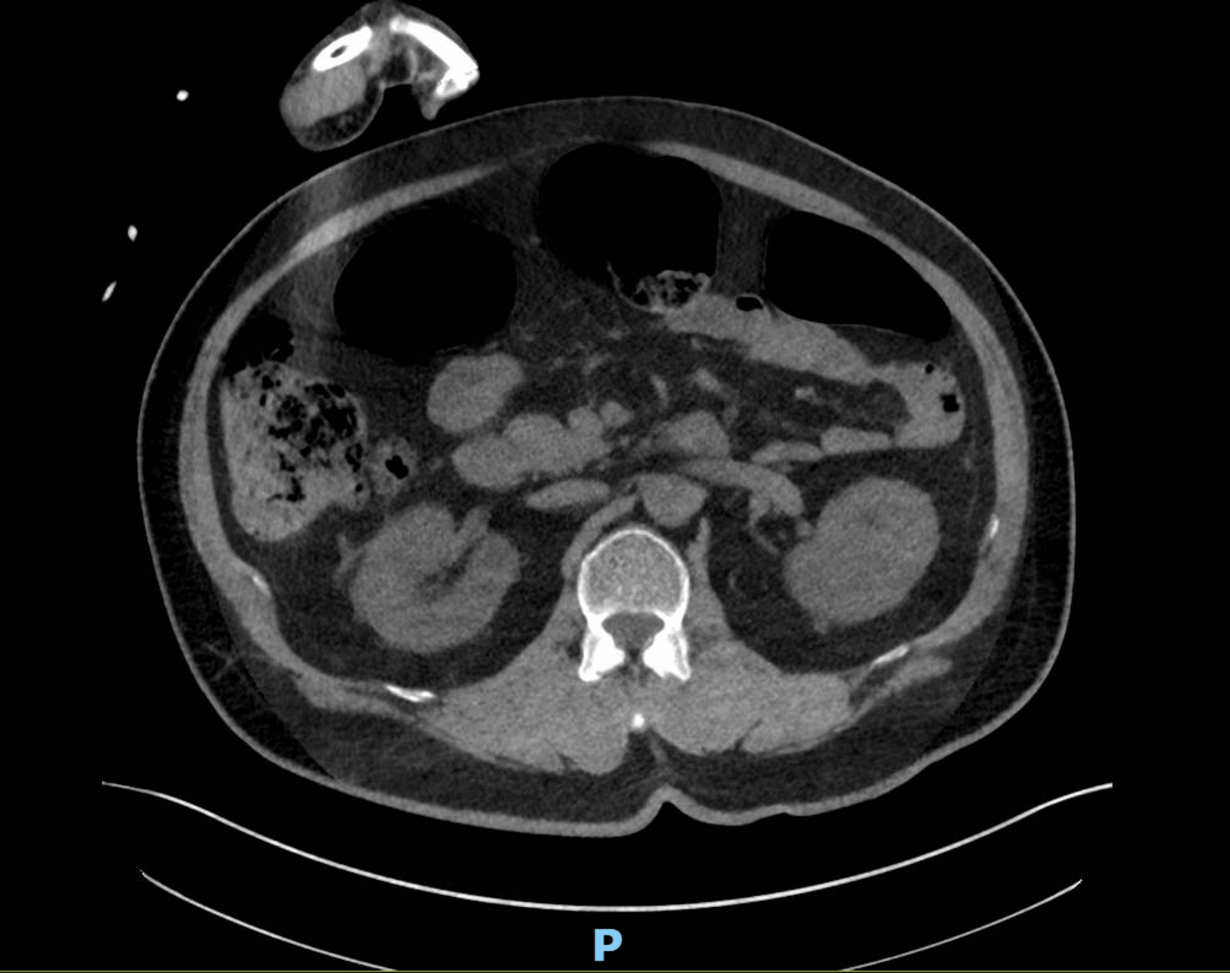
Contrast-enhanced CT of the thorax, abdomen, and pelvis (CT TAP). No evidence of malignancy or source of infection in the abdominal region.

**Figure 2:**
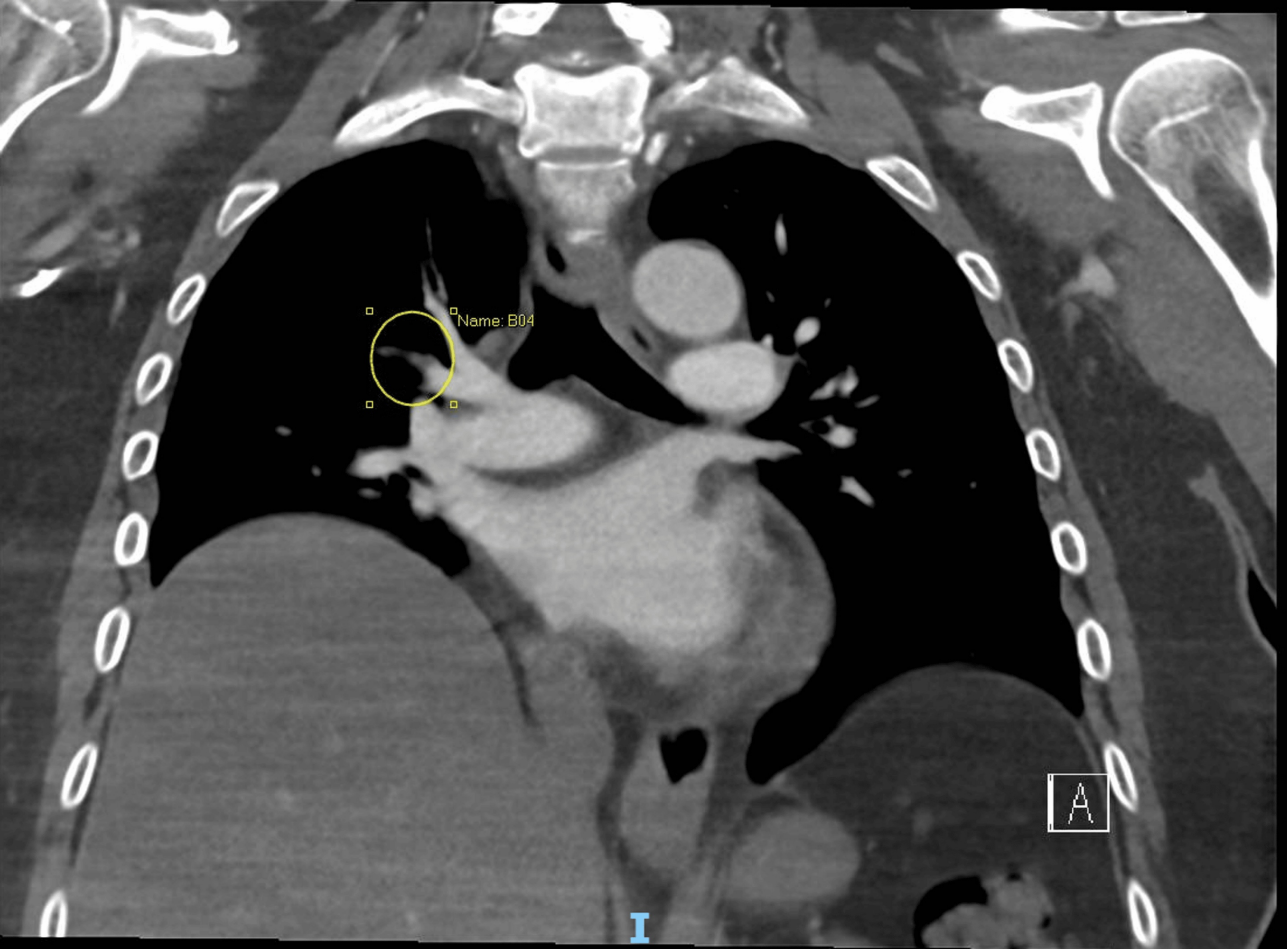
Computed tomography pulmonary angiography (CTPA), coronal view. Filling defects were noted around the subsegmental branches of the pulmonary arteries in the upper lobes of the lungs (yellow circle).

**Figure 3:**
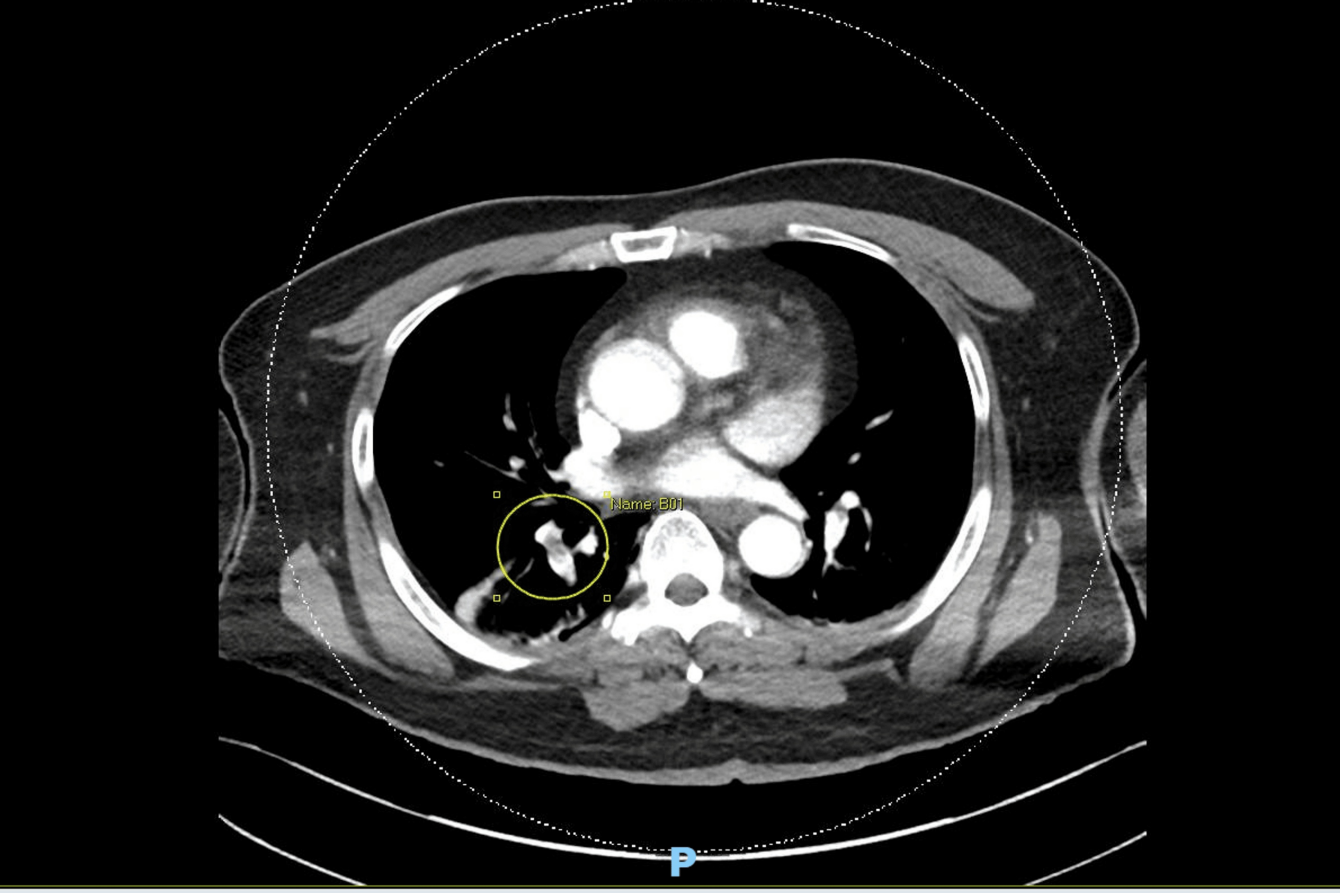
Computed tomography pulmonary angiography (CTPA). Filling defects were noted around the segmental branches of the pulmonary arteries in the lower lobes of the lungs (yellow circle).

**Figure 4:**
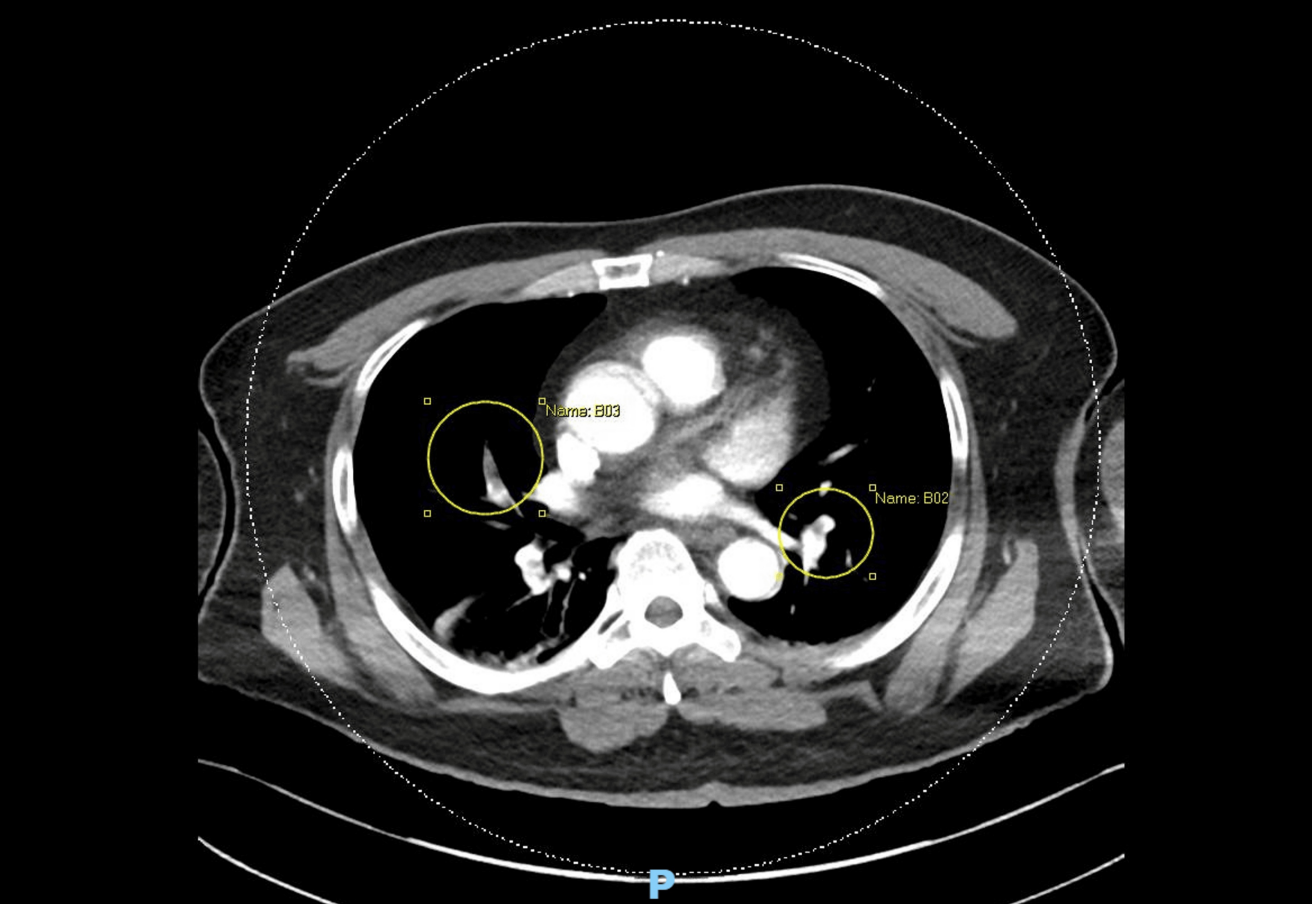
Computed tomography pulmonary angiography (CTPA). Filling defects were noted around the subsegmental branches of the pulmonary arteries in the middle lobes of the lungs (yellow circles).

Extensive investigations were pursued for persistent fever. Inflammatory markers (CRP and procalcitonin) remained low. CT thorax with CT pulmonary angiogram, abdomen, and pelvis performed in view of persistent fever, which revealed bilateral pulmonary embolism in segmental and subsegmental arteries (Figures 1-4). Creatine kinase was markedly elevated at 11,270 U/L (56-336U/L).

Clinically, he exhibited mutism, immobility, stupor, and echophenomena. Further psychiatric evaluation noted elevated mood, reduced sleep, and increased goal-directed activity. On repeat neurological examination, the patient demonstrated rigidity, particularly in the hands and lower extremities, with preserved reflexes and no tremor or clonus. The absence of tremor, clonus, or hyperreflexia - findings that may be seen in serotonin syndrome or NMS - made these alternative diagnoses less likely. Malignant catatonia was suspected based on the constellation of findings. A psychiatric consultation was obtained. The patient’s Bush-Francis Catatonia Rating Scale (BFCRS) score was 13, with contributing items including immobility/stupor (1), mutism (1), staring (2), verbigeration (3), rigidity (1), automatic obedience (2), and autonomic abnormality (3).

Lorazepam was initiated, leading to gradual improvement. Aripiprazole was added subsequently. Remarkably, his mental status improved within 24 hours, and his fever resolved after lorazepam administration, supporting the diagnosis of malignant catatonia. His BFCRS score decreased from 13 to 5 within 24 hours of lorazepam treatment. He was discharged on day 21 with significant recovery.

## Discussion

Malignant catatonia is a diagnosis of exclusion. It represents the most severe form of catatonia and can be life-threatening if not promptly recognized and treated [8]. Catatonia is broadly classified into three subtypes: (a) retarded type, characterized by mutism, inhibited movement, posturing, rigidity, negativism, and staring; (b) excited type, marked by excessive, purposeless motor activity, restlessness, stereotypy, impulsivity, and agitation; and (c) malignant catatonia, which is distinguished by features such as fever, autonomic instability (e.g., labile or elevated blood pressure, tachycardia, tachypnea, diaphoresis), delirium, and rigidity [9].

According to the Diagnostic and Statistical Manual of Mental Disorders, Fifth Edition (DSM-5), catatonia is diagnosed when three or more of the following symptoms are present: immobility, stupor, excitement, mutism, posturing, echophenomena, speech or behavioral mannerisms, stereotypy, and staring [10]. In our case, the patient demonstrated immobility, stupor, mutism, echophenomena, speech mannerisms, stereotypy, and staring, thus fulfilling the diagnostic criteria for catatonia.

Initially, his presentation was attributed to symptomatic hyponatremia. However, despite correction of sodium levels, his mental status did not normalize. Given the presence of fever and altered mental status, encephalitis was considered, but lumbar puncture and MRI brain findings were unremarkable. NMS was also considered, as both NMS and malignant catatonia share features such as fever, tachycardia, tremulousness, elevated creatine kinase, and rigidity [11,12]. In this case, however, the absence of clonus, hyperreflexia, and no recent changes in antipsychotic medication made NMS less likely. There was no withdrawal or dose escalation of olanzapine or venlafaxine. NMS typically requires recent exposure to antipsychotics (within 72 hours), particularly with dose increases or abrupt withdrawal of dopaminergic agents [11-13].

The BFCRS was instrumental in assessing severity and monitoring treatment response. A score of 13 indicated significant catatonic symptoms. The BFCRS consists of 23 items, each scored from 0 to 3, with higher scores reflecting greater severity [14]. The presence of two or more core items - such as excitement, immobility, mutism, staring, or posturing - is suggestive of catatonia [9,15]. The patient’s psychiatric history, clinical features, and rapid response to lorazepam supported the diagnosis of malignant catatonia. Differentiating malignant catatonia from NMS is crucial, as treatment strategies differ. Benzodiazepines, particularly lorazepam, remain the first-line therapy, while ECT is reserved for refractory cases. Literature supports the use of lorazepam at doses up to 24 mg/day in severe presentations [2,15]. A positive response is typically defined as a 50% reduction in BFCRS score [16].

Complications of malignant catatonia may include acute kidney injury, respiratory failure, cardiac arrest, and thromboembolic events [2,11]. In this case, the patient developed bilateral pulmonary embolism and transaminitis, likely reflecting systemic complications of the catatonic state.

## Conclusions

This case illustrates the complexity of diagnosing malignant catatonia, particularly in patients presenting with fever and altered mental status, where multiple differential diagnoses must be considered. Despite initial attribution of symptoms to hyponatremia, persistent encephalopathy and fever necessitated further evaluation, ultimately leading to the recognition of malignant catatonia. Early identification is critical, as timely treatment with benzodiazepines can be lifesaving and significantly improve outcomes, as illustrated in this case. This case highlights the importance of maintaining a high index of suspicion for malignant catatonia in psychiatric patients presenting with unexplained fever and altered mental status.

Malignant catatonia is associated with significant systemic complications, including thromboembolic events, as demonstrated by the bilateral pulmonary embolism in this patient. This underscores the importance of vigilant monitoring, early mobilization, and venous thromboembolism (VTE) prophylaxis in managing such cases.
